# Healthy Foods as Proxy for Functional Foods: Consumers' Awareness, Perception, and Demand for Natural Functional Foods in Pakistan

**DOI:** 10.1155/2019/6390650

**Published:** 2019-05-02

**Authors:** Akhter Ali, Dil Bahadur Rahut

**Affiliations:** ^1^International Maize and Wheat Improvement Center (CIMMYT), CSI Complex, NARC, Park Road, Islamabad, Pakistan; ^2^International Maize and Wheat Improvement Center (CIMMYT), Texcoco, Mexico

## Abstract

Using comprehensive primary dataset collected from 400 respondents from all four major provinces of Pakistan, this study assesses consumers' knowledge, awareness, and perception regarding the use of functional foods. The empirical findings show that the majority of the consumers do not have information and knowledge about the functional foods in Pakistan. Hence, the frequency of consumption of functional food was low especially in rural areas. The result revealed that consumers with ill health were more eager to consume functional foods compared to healthier people. Besides health, the level of education and gender (female) of the respondent also play significant role in the acceptability and consumption of the functional foods in Pakistan. Geographically the people in the cities were more aware and willing to pay more for the functional foods as compared to people living in the villages. Majority of the consumers think that consumption of functional foods can help them to maintain good health, hence the policy makers' needs to create more awareness.

## 1. Introduction

The concept of functional food was first time used and introduced in Japan during the 1980s [1], and Japan is the first and only country which has specific regulatory approval procedure for functional foods [1–3]. Functional foods are those food products which provide essential nutrients needed for good health [4] and which potentially have a positive impact on human health besides providing the necessary nutritional requirements. Food is viewed as a product to enhance health and wellbeing, and producers are responding proactively by supplying new goods that meet these needs [5, 6].

Over the years, the consumption pattern of consumers is evolving [7]. Number of studies in the past have shown that consumers demand food that helps prevent disease, boost mental health, and improve the quality of life [2, 8–12]. Innovation in the area of functional food products is very intense [13]. The global demand for functional foods is growing at around 8% annually, and the current level of the demand for functional foods is more than US$180 billion [14–16]. Nonetheless, the precise market size is hard to estimate because of the lack of standard definition of functional food [17].

Globally the consumers require food that is healthy and protects them from different disease [10, 15]. A consumer is only likely to consider switching conventional with functional food if the latter is perceived as healthier in comparison to conventional (Bech-Larsen et al., 2001). The awareness of functional food is growing, and its demand is increasing even in developing countries. Several forces are propelling the demand for functional food; increasing health conscious, growing health cost and awareness about the value of functional food are the primary driver. Hence, in this paper, we analyzed the consumers knowledge, awareness, and perception about the functional foods in Pakistan (for the easy understanding the healthy food has been used as proxy for the functional food throughout in this paper).

## 2. Literature Review

The demand for functional food is ever growing which is driven by concern about health and increase in life expectancy. Consumers are more and more anxious about their health and foster extra care to their lifestyle and the healthiness of their diet (Szakaly et al., 2012). The surge in demand for functional foods may be due to the increasing cost of healthcare, increase in life expectancy, and the longing to increase the quality of life [18]. Hence, health is the one of the important elements in the research related to the behavior of the consumer to the functional food (Figueroa and Sánchez, 2004).

Consumers display favourable attitudes and strengthen their willingness to buy functional food when it is projected as healthy and have advantageous nutritional facts [19]. Regardless of negative consumer perceptions of transgenic foods, the functional foods, which uphold the health benefits to those who consume them, are normally perceived as positive. Thus, it stresses the significance of the right communication of these encouraging health benefits [20].

The consumption of functional foods are likely to decrease the risk of the chronic diseases (Block et al., 1992). In the past, studies have established that the awareness about functional foods plays a vital role in their choice and acceptance of functional food [21, 22]. Nowadays foods are not only intended to provide essential nutrient for human body but also to prevent nutrition-related diseases [15, 23].

For low-income household and individuals, the price may be a barrier to the consumption of healthy food (throughout the current paper also healthy food has been used as proxy for the functional foods) like functional food. Given the significance of quality food, consumers are willing to pay premium price for foods, which contributes to better health [24]. The willingness-to-pay (WTP) estimates have been used for long in economics as a demand-revealing indicator; it is a welfare measure that relates to the sum an individual is willing to pay to ensure the change in the quality of a product. In case of functional food, it is the amount a consumer is willing to pay for the health characteristics in food for the potential health benefit from consuming the product [25].

Although it is essential to estimate the future demand for functional food before supplying such foods to the market, it is difficult to assess the potential demand due to innovative character of functional food and hence the nonavailability of actual market data. Therefore, hypothetical and non-market valuations of unique functional foods by consumers are frequently used to acquire the needed information for pricing and estimating the market [26, 27].

A significant section of the population search for functional food that offers larger benefit, reduces the risk of diseases, and supports good health. The presence of fiber in the food, high protein content, the presence of protein and minerals, and fortified food with calcium contents are regarded as the essential components of functional food. A large number of consumers place greater importance to sustainably produced food such as organic food. The consumer preference for functional food and willingness to pay for it arise from the fact that it contains local herbs and ingredients. Consumers with inadequate or small disposable income also desire to buy such food products that aid in meeting crucial nutritional requirements and are very appealing [28].

In most developed countries, the markets for functional foods have been increasing at great speed over the last two decades (in developing countries like Pakistan, the concept of functional food is still quite new). As the functional food provides health benefit along with providing basic nutrition, the market's share of functional foods in European countries was less than 1 percent in 2000 and it is expected to rise up to US$ 190 million in 2020 (Kaur, 2017). Similarly, the functional food is becoming more common in all the states of America with the passage of time. This growing use of functional foods is because consumers are becoming aware of the correlation between health and food that one eats. In addition to the awareness, the developments in the functional foods market combined with advances in food technology and nutritional sciences are playing an important role in fueling the functional foods [29].

In the past most of the studies on functional foods have been carried out in the developed world, i.e., Gilbert [30]; Frewer et al. [31]; Verbeke [32]; Jovanovic, [33]; O'Doherty et al., (2006); Kapolna and Lugasi, (2008); Paulionis, (2008); Vella et al., (2014); Boluda et al., [34]; Vassallo et al. (2009); Chen, (2011); Urala and Lahteenmaki (2007); Dolgopolova et al., [27]; Veneziani et al., [35]; Pasquale et al., [36]; Bechtold (2013) and not many studies have focused on the developing countries. The current study has many novel aspects; to the best of our knowledge, current study is the first which focuses on the perception of consumers about functional food in Pakistan. Secondly it also assesses the determinants and willingness to use functional food and thirdly, it estimates the impact of the functional food on human health which has been the focus of very few studies in the past. For that, the rest of the paper is organized as follows: in Section 3 data and sampling are presented; in Section 4 descriptive statistics is presented; in Section 5 empirical results are presented and the paper concludes in Section 5 with some policy recommendations.

## 3. Data and Sampling

For the current study, detailed data set was collected through field survey. Comprehensive questionnaire was used for the data collection. Information on a number of aspects was collected, specifically knowledge about functional foods, willingness to pay for the functional foods, and consumers' perception regarding health benefits of the functional foods. The data was collected from all the four major provinces of Pakistan, i.e., Punjab, Sindh, KPK, and Balochistan. In total data was collected from 400 respondents from both the urban and rural areas (before starting the formal survey, the pretesting of the questionnaire was carried out and the questionnaire was modified in the light of pretesting results). A team of well-trained enumerators carried out the survey during July-September, 2017. Before the start of the survey, the permission of the respondents was sought to participate in the survey. The selection of the respondents was made at random. The distribution of the respondent by province wise and rural and urban categories wise is summarized in Table 1. In the questionnaire, the definition used for the functional foods was “Functional foods are the foods that potentially have positive effect on health beyond basic nutrition”. Following this definition, the healthy foods, e.g., fruits, vegetables, and condiments, having positive impact on the human health were taken as proxy for the functional foods for the understanding of the consumers.

## 4. Descriptive Statistics

### 4.1. Description of Variable

Table 2 presents the description of variables used in the empirical analysis. The mean age of the respondents was 42 years and average years of schooling were about 13 years. About 63% of the respondents were male and the rest 37% were females. In the survey, the number of male respondents was more as compared to female because the enumerator team consisted of males enumerators who only hence were able to collect information from female who agreed to be part of the survey and also were comfortable to speak with male enumerators. Only 59% of the respondents were married. The average family size was about eight family members with 5 children per household and 38% of the respondents were living in the joint family system. About 50% of the respondents were living in the urban areas and the rest 50% were living in the rural areas (therefore, survey was good representative of the urban and rural areas).

The average household income was about 29,364 Pakistani rupees per month. Majority of the respondents were doing job, i.e., 71 percent; mostly the respondents in the urban areas were engaged in wage employment while the respondents in the rural areas were engaged in the agricultural activities including off farm labour work. Majority of the household owned television, i.e., 82%, about 69% of the respondent owned the house, and only 27% of the respondents owned the car. Majority of the respondents, i.e., 88 %, reported they were in good health.

In the questionnaire also information regarding the constraints in the functional foods was collected. About 73 percent of the respondents reported that price is a barrier in the purchase of the functional food while 36 percent of the respondents think that taste is the barrier in the use of the functional food. Majority of the respondents, i.e., 82 percent, have access to Internet facility (Through Internet the consumers can get information about various foods.).

### 4.2. Knowledge and Perception about Functional Food

**Figure 1:**
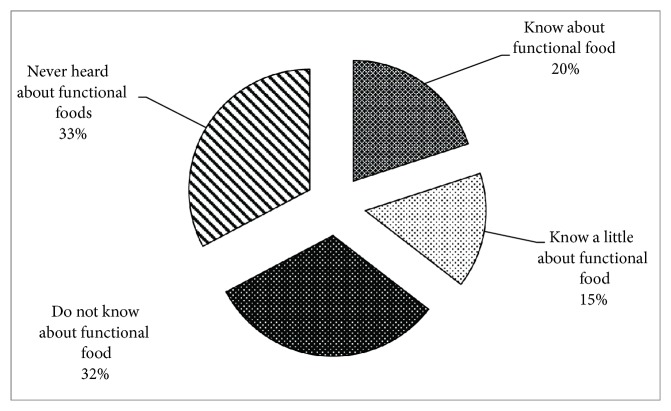
Consumers knowledge level about functional foods.

The detailed information about knowledge and perception of the functional foods is presented in Figure 1. Only about 20 percent of the respondents knew about the functional while the other 15 percent have little knowledge about the functional foods. The results indicate that 32% of the respondents did not know about functional food and 33% had never heard about functional food, which illustrates that the idea about functional food is still evolving and large population in Pakistan are not aware of functional food.

**Figure 2:**
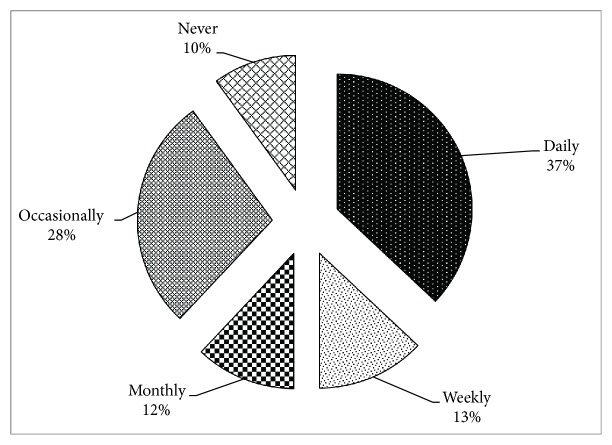
Frequency of functional foods.

The consumers' frequency of the consumption of the functional foods is presented in Figure 2. The results in Figure 2 indicate that about 37 percent of the respondents consume functional foods daily while 13 percent consume weekly and 12 percent consume monthly and 28 percent consume occasionally while the other 10 percent have never consumed. As majority of the consumers have no idea about the functional food, hence this was also really challenging to explain the idea of functional food here; the healthy food, i.e., fruits and vegetables, has been taken as proxy for the functional foods.

**Figure 3:**
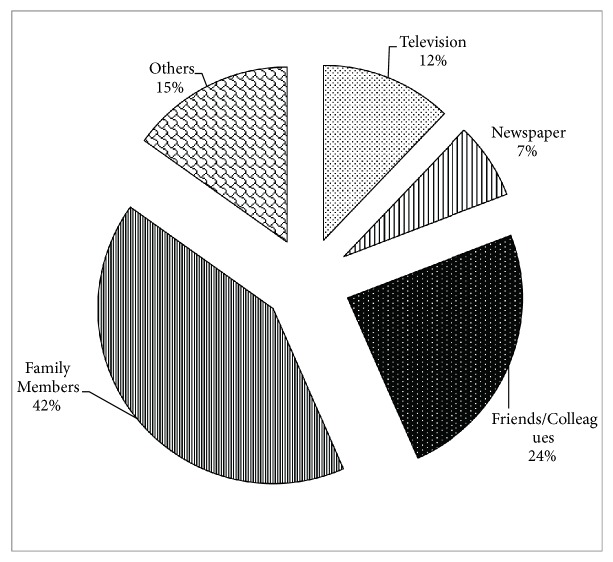
Source of information/awareness about functional foods.

The details about the source of the information are presented in Figure 3. Among the respondents who were aware about the functional food, about 42 percent came to know functional food from family members, 24 percent from friends and colleagues, 12 percent through television, and 15 percent from other sources. From the analysis, we can conclude that friends and family were the most important and key sources of information about functional foods.

**Figure 4:**
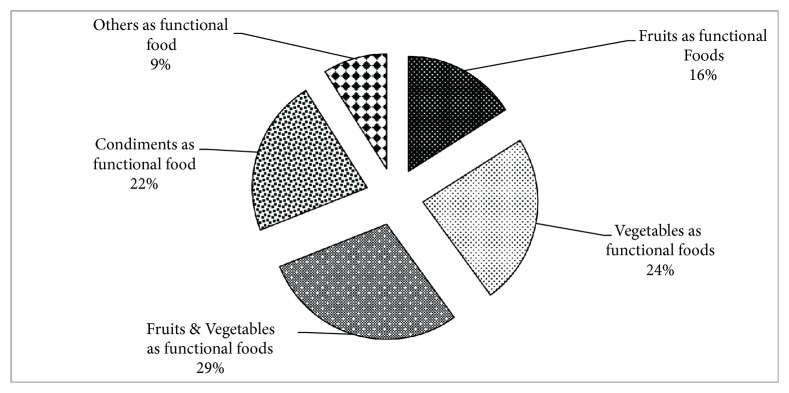
Sources of functional foods.

The individual sources of the functional foods are presented in Figure 4. The results indicate that 29 percent of the respondents consider fruits and vegetables as functional foods and 24 percent consider vegetables as functional food while 22 percent consider condiments as functional food, 16 percent consider fruits as functional food, and 9 percent consider others as functional food.

## 5. Empirical Analysis

### 5.1. Determinants of Awareness and Demand for Functional Food

Number of factors including social and cultural factors [37, 38] affects the awareness and acceptance of the functional foods. Bivariate probit model was used to estimate the awareness and demand of functional food. As the dependent variable is dummy, i.e., 1 if the respondent is aware about the functional food and 0 otherwise, the second dependent variable is the demand for the functional food, 1 if the consumer demand functional food and 0 otherwise. A set of independent variables was included in the model, i.e., demographic, human capital, financial capital, and access to market, etc., which were included in the model and the results are presented in Table 3.

The age coefficient was negative and nonsignificant indicating that age does not really matter regarding the awareness and demand for the functional food. The gender was included as dummy variable (i.e., 1 for the male and 0 for female) and the coefficient was negative and significant indicating that females were likely to have higher level of awareness and knowledge about functional foods and are also likely to consume more functional foods. This is in line with the fact that females mostly are more concerned about their health. The gender results are inline with the previous studies as in the past a number of studies have found that females were more aware about the functional foods and also were willing to pay more for the function foods [39–42].

The marital status was included as dummy variable (married is equal to 1 and 0 otherwise) and the coefficient is positive and nonsignificant. The family size coefficient was positive for awareness and demand of the functional foods, but it was significant only for awareness of functional food. As the larger family size provides more social network which increases the chances of awareness, the dependency ratio, i.e., number of children in the household coefficient, was negative but significant only for demand of functional food indicating that more dependency ratio leads to lower demand for functional food.

The coefficient of years of education of respondent was positive and highly significant indicating that educated respondents were more aware and were willing to consume more functional foods. The wage coefficient was positive and significant regrading awareness of the functional food while negative and significant regarding demand of the functional food. The coefficient of respondent's income was also positive and highly significant for both awareness and demand for functional food, which indicates that wealthy households were more aware about the functional foods. Similarly, the house ownership and television ownership were positive and significant on awareness and demand for functional food. Hence, we can conclude that richer individuals and families were more aware about functional food and also consume more as compared to poor individuals.

If the respondents perceive that they are in good health, they are less likely to be aware of the functional food, which is obvious as healthy individuals do not search or explore for healthy foods (functional food). However, healthy person seems to consume more of functional food. The price of functional food increases its level of awareness because consumers perceive high priced commodity as the high quality product. However, the demand and price of the functional foods are inversely related. The taste of the functional food is positively associated with the demand for it.

The coefficient of location dummy variable (i.e., 1 for the urban and 0 for the rural) was positive and significant indicating that respondents from the urban areas were more aware about the functional food and were also more likely to demand more functional food as compared to respondents of the rural areas.

The determinants of the factors influencing the number of functional foods (due to lack of awareness, the healthy food has been used as proxy for the functional food) consumed per week are presented in Table 4. For the number of healthy foods/functional foods, the Poisson regression model has been estimated, and the dependent variable is the number of functional foods consumed per week. During the interviews per day information was collected and that per day information was converted to weekly basis. The healthy food, i.e., consumption of the fruits and vegetables, has been used as proxy for the functional foods. All the information was based on the respondents recall/memory and the educated respondents were able to provide more robust responses as compared to less educated respondents but as the data was collected randomly, hence the respondents were a mix of both educated and less educated respondents. Result shows that female respondents were more likely to consume more number of functional foods as women were more concerned about the health and wellbeing of the family member. The coefficient of the number of years of schooling was positive and significant at 1% level of significance indicating that the respondents with higher number of years of schooling were likely to consume more number of functional foods.

The higher the income, the greater the number of functional foods consumed because income level indicates the affordability. Wage employed people were also likely to consume more number of functional foods compared to those employed in agriculture. Those respondents with television ownership were also consuming more number of functional foods because it brings about the awareness about the functional food. From this analysis, we can confirm that wealth/income positively influences the number of functional foods consumed per week.

The coefficient of the dummy of good health is negative and significant highlighting the fact that healthy individuals consume less number of functional foods. The urban dummy is positive and significant at 1% level of significance signifying that respondents from the urban area were more likely to consume more number of functional foods compared to the rural respondents.

### 5.2. Preferred Sources of the Functional Food

In Pakistan beside fruits and vegetables the condiments were also the preferred sources of the functional foods. For the consumers' preferred sources of functional food, multivariate probit model was estimated and the results are presented in Table 5. The dependent variables are the sources of the functional food, i.e., fruits, vegetables, condiments, and others (pulses, cereals); hence for that multivariate probit model is the most suitable model. The cross-equation correlation (Table 6) also confirms the suitability of the multivariate probit model.

The age of the household head was positive and significant for condiments indicating that aged people prefer condiments as the functional food. The gender coefficient was negative and significant indicating that female respondent prefers more of fruits, vegetables, and condiments as functional foods as compared to males while male respondents prefer more of other functional foods, i.e., cereal and pulses, etc. Women acceptance of the functional foods is inline with the previous studies, e.g., Monneuse et al. [43] and Bogue et al. [44]. The marital status, family size, family system, and children were mostly nonsignificant.

The coefficient of years of education was positive and significant for all types of functional food except for others indicating that educated respondents prefer more of fruits, vegetables, and condiments as functional food as compared to less educated respondents. The positive association of preferred functional food and education has been confirmed by the previous studies, e.g., Jong et al. [45].

The coefficient of the household income is positive and significant for all types of functional food (i.e., fruits, vegetables, condiments, and others), which shows the positive role of the income on preference and consumption of the functional food. Similarly, the household assets particularly television and house ownership was positive and significant indicating that wealthy households mostly use the functional food. Previous studies show that wealthy individual prefers and consumes more of the functional foods as compared to poor individuals (Bogue et al. (2003).

The health status dummy was positive and significant for vegetables and condiments indicating that respondents in good health mostly use the functional foods such as vegetables and condiments while it was negative and significant for fruits.

The price coefficient was negative and significant for price of fruits indicating that higher prices lead to less use of fruits while it was positive and significant for vegetables and condiments (this contradicts to economic theory but mostly the prices of vegetables and condiments are stable as compared to fruits). The taste was positive but significant only for condiments indicating good taste of condiments positively influences the consumption of condiments. Higher access to Internet tends to lead to more preference of functional food and vice versa. The location coefficient was positive and significant indicating that the respondents living in urban areas mostly consume the functional foods as compared to respondents living in the rural areas.

The LR chi square (≤0.001) is highly significant at 1 percent level of significance indicating robustness of variables included in the model.

### 5.3. Consumers' Willingness to Pay (WTP) for the Functional Food

The censored least absolute deviation (CLAD) model was estimated regarding consumers' willingness to pay for the functional food and the results are presented in Table 7. The dependent variable was the amount of money consumers were willing to pay for the functional food. The health benefits of the functional foods were explained to the respondents and they were asked how much money they were willing to pay for the functional food.

The age coefficient was negative and significant indicating that mostly the young respondents were willing to pay for the functional foods compared to the older respondents. Younger respondents were willing to pay more for functional food as compared to aged respondents as they were more aware of the value of functional food on health. The gender dummy (male respondent) was negative and significant which implies that females were more willing to pay for the functional food compared to male respondents. Previous studies have shown that females have greater knowledge as well as the intention to use functional food [18, 46]. The marital status, family size, family system, and children coefficients were non-significant.

The year of schooling was positive and highly significant, which is as expected because with the increase in the level of education individuals were more aware of the importance of functional food on health. The positive association between education and willingness to pay for the functional food arises from the fact that respondent with higher education has more knowledge and intention to pay for the function food. The results are in line with Siro et al. [18] and Hilliam [47].

The income, wage employment, and household assets coefficients (such as television, house ownership, and car) were positive and significant implying that the wealthy households were more willing to pay for the functional foods. The respondents' health status is negative and significant, hence indicating that respondents with good health were more willing to pay for the functional foods. The coefficients for the price, taste, and Internet were all positive and nonsignificant. The location coefficient was positive and significant indicating that respondents in the cities were more using functional.

The LR chi-square (≤0.001) was positive and highly significant at 1 percent level of significance indicating the robustness of variables included in the model. The initial number of observations was 400, and the final number of observations was 316.

### 5.4. Health Impact of Functional Food

Consumer's perception on the impact of functional food has been estimated by employing the propensity score matching estimates. In case of PSM two different matching algorithms, i.e., Radius matching (RM) and Splene matching (SM), were employed. The respondents perception was estimated on overall health status. The results are presented in Table 8.

The empirical results show that respondent's perception on the overall health status was positive and significant indicating that respondents consider functional good for human health.

The past studies have shown that consumers perceived benefits of the functional foods were very important factors regrading consumers acceptance of the functional foods [32, 48, 49]. After matching the quality of matching was also checked by employing a number of robustness tests. The results of matching are inline with previous studies [50, 51].

## 6. Conclusion

The consumers in Pakistan mostly lack information and awareness about the functional foods. However the consumers living in urban areas were more awared as compared to consumers living in rural areas. The consumers wealth status, education level, and gender play a significant role regarding acceptability of functional foods in Pakistan. The educated respondents have more knowledge and were prepared to pay more for the functional foods. The gender is another important indicator; i.e., females were more prepared to accept the functional foods as compared to males.

The propensity score matching estimates indicated that the respondents using functional foods have less diseases and have good health. However the lack of awareness is the main issue regrading promotion of the functional foods. The sick people were willing to pay more for the functional food as compared to healthy people.

The consumers' willingness to pay analysis indicated that wealthy consumers having higher assets ownership were more willing to pay for the functional foods. Similarly the respondents in cities were more willing to pay for the functional foods as compared to living in rural areas. The policy implications suggest that more awareness needs to be created through media campaign.

## Figures and Tables

**Table 1:** Distributions of the respondents by province, rural, and urban areas.

		No. of respondents		
	No. of districts	Urban	Rural	Total
Total	26	203	197	400
Punjab	13	102	98	200
Sindh	6	47	45	92
KPK	5	39	37	76
Baluchistan	2	17	15	32

**Table 2:** Data and description of variables.

Variable	Description	Mean	Std. Dev
*Demographic*			
Age	Age of the respondent in numbers of years	41.64	6.84
Gender (dummy)	1 if the respondent is male, 0 otherwise	0.63	0.55
Marital status (dummy)	1 if the respondent is married, 0 otherwise	0.59	0.42
Family size	Number of family members living in the household	8.24	6.34
Children	Number of Children living in the household	5.13	3.16
Family system (dummy)	1 if living in joint family, 0 otherwise	0.38	0.29
*Human capital*			
Education	Years of schooling of the respondent	12.55	5.27
*Income & employment*			
Income	Average Income of the household in rupees	29364	1764
Job (dummy)	1 if the respondent is doing job, 0 otherwise	0.71	0.56
*Wealth*			
Television (dummy)	1 if the respondent owns a TV, 0 otherwise	0.82	0.67
House (dummy)	1 if the respondent owns a House, 0 otherwise	0.69	0.34
Car (dummy)	1 if the household owns a Car, 0 otherwise	0.27	0.21
Health condition			
Health status (dummy)	1 if the respondent is in good health, 0 otherwise	0.88	0.68
*Perception on price and taste*			
Price	1 if the respondent thinks that price is a barrier in the purchase of functional food, 0 otherwise	0.73	0.56
Taste	1 if the taste prevents the use of functional food, 0 otherwise	0.36	0.24
*Access to information*			
Internet	1 if the respondent has access to the Internet, 0 otherwise	0.82	0.37
*Location*			
Urban	1 if the respondent is living in urban areas, 0 for rural areas	0.5	0.42

**Table 3:** Determinants of functional foods awareness and demand (Bivariate probit model).

Variable	Functional Food Awareness		Functional Food Demand	
	Coefficient	t-vales	Coefficient	t-values
*Demographic*				
Age	-0.06	-1.19	0.02	1.32
Gender	-0.03*∗*	-1.72	-0.08*∗∗*	-1.99
Marital Status	0.04	0.87	0.11	1.35
Family size	0.01*∗∗*	2.19	0.07	1.62
Family system	0.03	1.27	0.05	1.58
Children	-0.09	-1.44	-0.06*∗*	-1.83
*Human capital*				
Education	0.02*∗∗*	2.14	0.14*∗∗∗*	3.45
*Income & employment*				
Income	0.06*∗*	1.94	0.04*∗∗∗*	2.89
Wage employment	0.11*∗*	1.77	-0.08*∗*	-1.82
*Wealth*				
Television	0.03*∗∗*	2.06	0.004*∗∗∗*	2.50
House	0.07*∗∗*	2.13	0.05*∗∗*	2.14
Car	0.02	1.05	0.06	1.34
*Perception on health status, price, and taste*				
Health status	-0.06*∗∗*	-2.18	0.05*∗*	1.92
Price	0.14*∗*	1.21	-0.04*∗∗*	-2.15
Taste	0.04	1.35	0.03*∗*	1.92
*Access to information*				
Internet	0.05*∗∗*	2.17	0.01*∗∗*	2.06
*Location*				
Urban	0.01*∗∗∗*	2.73	0.02*∗∗*	2.19
Constant	0.04*∗*	1.90	0.03	1.23
LR Chi-Square	284.27			
Prob>Chi Square	≤0.001			
Value of R-square	0.27			
Numbers of Observations	400			

Note: Results are significant at *∗∗∗*, *∗∗*, *∗* 1, 5, and 10 percent levels, respectively.

**Table 4:** Numbers of the functional food items consumed per week (Poisson regression estimates).

Variable	Coefficient	t-values
*Demographic*		
Age	0.03	1.54
Gender (male)	-0.01*∗*	-1.85
Marital Status	0.11	1.36
Family size	-0.02	-1.40
Family system	-0.01*∗*	-1.73
Children	0.04	1.29
*Human capital*		
Education	0.08*∗∗∗*	3.02
*Income and employment*		
Income	0.02*∗∗∗*	3.19
Job	0.07*∗∗*	2.08
*Wealth*		
Television	0.03*∗*	1.93
House	0.05	0.83
Car	0.01	1.55
*Perception on health and the price and taste of functional food*		
Health status	-0.08*∗∗*	-2.11
Price	0.06	1.42
Taste	0.03	1.36
*Access to information*		
Internet	0.13	1.52
*Location*		
Urban	0.09*∗∗*	2.05
Constant	0.08*∗∗*	2.04
LR Chi-Square	153.28	
Prob>Chi Square	≤0.001	
Value of R-square	0.31	
Numbers of Observations	400	

Note: Results are significant at *∗∗∗*, *∗∗*, *∗* 1, 5, and 10 percent levels, respectively.

**Table 5:** Preferred source of functional food (multivariate probit model).

Variables	Fruits		Vegetables		Condiments		Others	
	Coefficient	t-values	Coefficient	t-values	Coefficient	t-values	Coefficient	t-values
*Demographic*								
Age	-0.05	-1.28	0.07	1.42	0.01*∗∗*	2.15	0.04	1.23
Gender	-0.03*∗*	-1.76	-0.04*∗∗*	-2.11	-0.06*∗*	1.80	0.09*∗*	1.72
Marital Status	0.01	1.47	0.02	1.57	0.03	1.36	0.08	1.62
Family size	0.08	1.34	0.13	1.63	0.05	1.09	0.10	1.47
Family system	0.03	1.51	0.07	1.28	0.04	1.20	0.09	1.53
Children	-0.05	1.43	0.06	1.49	0.05*∗*	1.83	0.04	1.02
*Human capital*								
Education	0.02*∗*	1.93	0.05*∗*	1.68	0.14*∗∗∗*	2.96	0.02	1.46
*Income and employment*								
Income	0.01*∗∗∗*	3.17	0.04*∗∗*	2.16	0.05*∗*	1.92	0.04*∗*	1.73
Job	0.05	1.26	0.04	1.08	0.06	1.48	0.01	1.28
*Wealth*								
Television	0.03*∗*	1.91	0.02*∗*	1.95	0.04*∗∗*	2.16	0.09*∗*	1.68
House	0.04*∗∗*	2.18	0.05	1.72	0.06*∗*	1.75	0.01	1.39
Car	0.06*∗*	1.73	-0.01	-1.57	0.03	1.29	0.04	1.10
*Perception on health and the price and taste of functional food*								
Health status	-0.02*∗∗∗*	2.57	0.03*∗∗*	2.12	0.11*∗∗∗*	3.06	0.05	1.25
Price	-0.01*∗∗*	2.34	0.07*∗*	1.93	0.10*∗*	1.83	0.04	1.43
Taste	0.03	1.52	0.04	1.61	0.07*∗*	1.67	0.03	1.54
Access to information								
Internet	0.06*∗*	1.73	0.05	1.38	0.04	1.20	0.05*∗*	1.81
*Location*								
Urban	-0.06*∗*	-1.30	-0.05*∗∗*	-2.14	-0.004*∗∗*	-1.98	0.16*∗∗*	2.76
Constant	0.04*∗*	1.84	0.04	1.76	0.05*∗*	1.78	0.04	1.52

LR Chi Square	340.94							
Prob>Chi Square	≤0.001							
Value of R-square	0.36							
Numbers of Observations	400							

Note: Results are significant at *∗∗∗*, *∗∗*, *∗* 1, 5, and 10 percent levels, respectively.

**Table 6:** Cross equation correlations.

Dependent variable	Cross equation correlations
Fruits and Vegetables	0.135*∗∗∗*(2.51)
Fruits and Condiments	0.243*∗∗*(2.08)
Fruits and Others	0.169*∗*(1.76)
Vegetables and Condiments	0.207*∗∗*(2.34)
Vegetables and Others	0.255*∗∗∗*(2.94)
Condiments and Others	0.162*∗∗*(2.37)

**Table 7:** Consumers' willingness to pay for the functional foods (Tobit estimates).

Variable	Coefficient	t-values
*Demographic*		
Age	-0.01*∗*	1.23
Gender	-0.02*∗∗*	2.34
Marital Status	0.04	1.33
Family size	-0.03	-1.78
Family system	0.05	1.44
Children	-0.03	-1.23
*Human capital*		
Education	0.07*∗∗*	2.16
*Income and employment*		
Income	0.04*∗∗*	2.24
Job	0.03	1.45
*Wealth*		
Television	0.13*∗∗*	2.18
House	0.06*∗*	1.93
Car	0.02*∗∗*	2.76
*Perception on health and price and taste of functional food*		
Health status	-0.01*∗*	-1.82
Price	0.04	1.30
Taste	0.03	1.28
*Access to information*		
Internet	0.04	1.55
*Location*		
Urban resident	0.12*∗∗∗*	2.51

LR Chi Square	294.34	
Prob>Chi Square	≤0.001	
Value of R-square	0.19	
Numbers of Observations	400	
Censored Observations	84	
Final Observations	316	

Note: Results are significant at *∗∗∗*, *∗∗*, *∗* 1, 5, and 10 percent levels, respectively.

**Table 8:** Consumers perception regarding impact of functional food on human health (PSM estimates).

Outcome	Caliper	ATT	t-values	Critical Level of	Numbers	Numbers
				Hidden Bias	of Treated	of Control
Matching Algorithm: Radius Matching (RM)						
Health	0.03	+++*∗∗∗*	3.14	1.45-1.50	235	117
						

Matching Algorithm: Splene Matching (SM)						
Health	0.06	+++*∗∗∗*	2.87	1.35-1.40	249	83
						

Note: ATT stands for the average treatment effect for the treated. RM stands for the radius matching while SM stands for the Splene matching. The results are significant at *∗∗∗* 1 percent levels, respectively.

## Data Availability

The data can be shared upon request.
